# *In Vitro* Alpha-Glucosidase Inhibitory Effect of *Etlingera Elatior* Ethanol Extract Growing in Gayo Highland, Aceh Province, Indonesia

**DOI:** 10.12688/f1000research.149029.2

**Published:** 2024-09-04

**Authors:** Zumaidar Zumaidar, Nuzul Asmilia, Saudah Saudah, Milda Husnah

**Affiliations:** 1 Department of Biology, Faculty of Mathematics and Natural Sciences, Universitas Syiah Kuala, Banda Aceh, Aceh, 23111, Indonesia; 2 Clinical Laboratory, Faculty of Veterinary Medicine, Universitas Syiah Kuala, Banda Aceh, Aceh, 23111, Indonesia; 3 Faculty of Teacher Training and Education, Universitas Serambi Mekkah, Banda Aceh, Aceh, 23245, Indonesia; 4 Graduate School of Mathematics and Applied Science, Universitas Syiah Kuala, Banda Aceh, Aceh, 23111, Indonesia

**Keywords:** Antidiabetic, anti-hyperglycemic, α-glucosidase inhibitor, antioxidant activity, Etlingera elatior

## Abstract

**Background:**

The prevalence of diabetes mellitus (DM) is increasing overtime, potentially leading to various severe health complications and mortality. Despite therapeutic agents have currently been developed, unexpected adverse effects are inevitable. Hence, safe and effective medications such as those of plant origin are critical to prevent unexpected complication in DM sufferers. *Etlingera elatior* has been widely used as spice and traditional medicine to treat diabetes in Aceh Province, Indonesia. However, study regarding α-glucosidase inhibitory effect of *E. elatior* growing in Gayo highlands, Aceh, Indonesia, is completely lacking. The aim of this study was to evaluate *in vitro* α-glucosidase inhibitory effect of *E. elatior* ethanol extracts (EEEE) growing in Gayo highlands, Aceh Province, Indonesia.

**Methods:**

Antioxidant activity was determined using DPPH procedure, whereas α-glucosidase inhibition assay was carried out using spectrophotometric method. Data analysis was performed using One-Way Analysis of Variance (ANOVA), followed by Duncan’s multiple range test at α=0.05.

**Results:**

Phytochemical analysis revealed the presence of total phenolic (TPC), total flavonoid (TFC), and total tannin (TTC) content in all *E. elatior* plant parts, in which the highest TPC was found in the stem (158.38 GAE/g), whereas the highest TFC and TTC was obtained in the rhizome extracts. The extract of fruit showed the strongest antioxidant activities, followed by the stem and leaf, with IC _50_ of 2.381 μg/mL, 6.966 μg/mL, and 19.365 μg/mL, respectively. All *E. elatior* extracts revealed a significant inhibitory activity against α-glucosidase at the concentration of 500 μg/mL, in which the stem extract showed the most effective α-glucosidase inhibitory effect with IC _50_ value of 5.15 μg/mL, suggesting its promising potential as antidiabetic agent.

**Conclusions:**

This study highlights *E. elatior* potency as a novel source of antioxidant and natural antidiabetic compounds that are useful for the prevention and treatment of diabetes.

## Introduction

Diabetes mellitus (DM) has been considered one of the leading causes of death worldwide. ^1^ Approximately 415 million adults suffer from diabetes globally, and the number is estimated to increase up to 642 million by the year of 2040. ^2^ In the Southeast Asian regions, around 11.3% prevalence of DM has been reported and Indonesia occupies the 7th most DM sufferers, which is considered a major contributor of diabetes cases in Southeast Asia. ^3^ DM often leads to various health complications such as blindness, stroke, heart disease, and kidney failure. ^4^ According to the World Health Organization (WHO) report, around 422 million people suffer from DM at the age of 20-70 years worldwide. Several factors, including age, obesity, lifestyle, and unhealthy diets are among risk factors for diabetes. ^5^

Most of diabetic patients use oral preparations and insulin injections as therapy; however, taking long-term oral medications may result in unexpected adverse effects. ^6^^,^^7^ This condition has urged the need for searching and developing new therapeutic approaches that are safer and easier for users, ^8^ including the use of α-glucosidase inhibitors, especially those of plant origin. Αlpha-glucosidase inhibitors are oral antidiabetics that act by inhibiting α-glucosidase enzyme responsible for catabolizing complex polysaccharide into monosaccharides, causing a reduction in glucose absorption and postprandial blood sugar levels. ^9^

Identification of potent natural compounds effectively inhibiting α-glucosidase has been extensively carried out as an effort in searching for new antidiabetic agents. ^10^ Some α-glucosidase inhibitors have been isolated from medicinal plants, offering the potential for alternative medications with greater efficacy and fewer side effects compared to current treatments. ^11^ Among the traditionally favored source of alternative medicines for the treatment of diabetes is *Etlingera elatior.* ^12^ Research on *E. elatior* flower extract revealed a strong inhibitory activity against α-glucosidase, making it a promising candidate for diabetes treatment. ^13^ Rhizome of *E. elatior* was also reported to exhibit antidiabetic effect attributed to its flavonoid compounds. ^14^ However, there is currently lack of information regarding antioxidant and antidiabetic properties of *E. elatior* extracts growing in Gayo highlands, Aceh, Indonesia. Therefore, the aim of this study was to evaluate antioxidant and α-glucosidase inhibitory activities of *E. elatior* extracts growing in this specific region.

## Methods

### Materials

The materials used in this study included technical ethanol 96%, Folin-Ciocalteu’s reagent (Merck-1.09001.0100, Germany), sodium carbonate (Na _2_CO _3_) (Merck-1.06392.0500, Germany), gallic acid (Merck-8.42649.0025, Germany), sodium nitrite (NaNO _2_) (Merck-1.06549.0500, Germany), aluminium chloride (AlCl _3_) (Merck-8.01081.0500, Germany), sodium hydroxide (NaOH) (Merck-1.06498.1000, Germany), quercetin (Sigma Aldrich-Q4951, USA), vanillin (Merck-8.18718.0100, Germany), hydrochloric acid fuming (HCl) (Merck-1.00317.2500, Germany), tannic acid (Sigma Aldrich-1643328-2G, USA), 1,1-Diphenyl-2-picrylrazyl (DPPH) powder (Tokyo Chemical Industry Co., Ltd. (JP)-D4313, Japan), ascorbic acid (Merck-1.00468.0100, Germany), phosphate buffer saline (PBS) (Oxoid-BR0014G, England), dimethyl sulfoxide (DMSO) (Merck-1.02952.1000, Germany), α-glucosidase powder (CDH-105265, India), bovine serum albumin (Himedia-MB083-25G, India), p-nitrophenyl-D-glucopyranose (p-NPG) (Sigma-N1377-5g, USA), and acarbose.

### Sample collection

*Etlingera elatior* plant was obtained from Gayo Highlands of Aceh Province, Indonesia. The plant was identified and preserved at Acehense Herbarium, Department of Biology, Universitas Syiah Kuala, Indonesia. Based on the International Union for Conservation of Nature (IUCN), the plant is classified into “data deficient (DD)” species due to inadequate information for its conservation status assessment. Meanwhile, this plant widely grows in various areas in Aceh, including forest, garden, or at the backyard of Acehnese houses since it has been traditionally used as traditional medicine, spice, and food. ^15^

### Extracts preparation

Samples of *E. elatior* plant parts consisting of rhizomes, stems, leaves, flowers, and fruits were washed thoroughly under running tap water. The samples were then air-dried for 6 days and diligently grinded to obtain powder. Extraction was carried out by the maceration method. In brief, a 100 gram of simplicial was placed into a maceration container, filled with ethanol 96% until submerged, homogenized, sealed, and let stand for 24 hours with occasional stirring. The mixture was then filtered yielding macerate and lees. The macerate was stored and the lees underwent the same maceration procedure until the solvent became colourless. The obtained macerate was evaporated by vacuum rotary evaporator (Buchi R-300, Swiss) at 50°C to obtain concentrated (100%) stem, leaf, fruit, flower, and rhizome extracts. ^16^ The extracts were then stored at 20°C for further analysis.

### Total Phenolic Content (TPC)

Each EEEE samples (stems, leaf, fruit, flower, and rhizome) (0.2 mL) with a concentration of 300 μg/mL was mixed with 2.5 mL of 10% (v/v) Folin-Ciocalteu’s reagent and 2 mL of 7.5% (v/v) Na _2_CO _3_. After being incubated for 10 minutes at 45°C, the absorbance of the solution was measured using a UV-Vis spectrophotometer at a wavelength of 765 nm (UV-1800, Shimadzu, Japan) for the determination of TPC. Standard solution of gallic acid (concentrations of 10 – 100 μg/mL; R ^2^= 0.9237) was used to make a calibration curve, and TPC was expressed as mg (+)- of gallic acid equivalent per gram of dry weight of the extracts (GAE/g). ^17^^,^^18^ The assay was performed in triplicate.

### Total Flavonoids Content (TFC)

Each extract sample (5 mL) with a concentration of 500 μg/mL was mixed with 0.3 mL of 5% (v/v) NaNO _2_ and 0.3 mL of 10% v/v AlCl _3_. After incubation for 5 minutes, 2 mL of 1 M NaOH was added to the concoction. The absorbance of the solution was measured using UV-Vis spectrophotometer at a wavelength of 510 nm (UV-1800, Shimadzu, Japan). The calibration curve of standard quercetin (concentrations of 10 – 400 μg/mL; R ^2^= 0.9282) was used for quantification of TFC in the extracts, which was expressed as mg (+)- quercetin equivalent per gram of dry extract (QE/g). ^18^^,^^19^ The assay was performed in triplicate.

### Total Tannin Content (TTC)

Condensed tannins (proanthocyanidins) were determined by mixing 50 μL of the diluted EEEE sample with 3 mL of 4% vanillin in methanol and 1.5 mL of concentrated HCl. The mixture was allowed to stand for 15 minutes and the absorption was measured using UV-Vis spectrophotometer (UV-1800, Shimadzu, Japan) at a wavelength of 500 nm with respect to methanol as a blank control. Quantification of total tannin in the extracts was based on tannic acid (concentrations of 1.5 – 20 μg/mL; R ^2^ = 0.9705) calibration curve, and the total amount of condensed tannins was expressed as mg (+)- tannic acid equivalent per gram of dry extracts (TAE/g). ^18^^,^^20^ The assay was performed in triplicate.

### 1,1-Diphenyl-2-picrylrazyl (DPPH) assay

DPPH assay was employed to assess free radical-scavenging activity of EEEE. Briefly, 3.5 mL of DPPH solution and 0.5 mL of each sample extract (concentrations of 1000, 500, 250, 125, 62.5, and 31.5 μg/mL) were mixed, homogenized, and incubated at 27°C for 20 minutes. The absorbance of the solution was then measured at a wavelength of 517 nm using UV-Vis spectrophotometer (UV-1800, Shimadzu, Japan). The ascorbic acid of various concentrations (2, 4, 6, and 8 μg/mL) was used as the positive control. ^19^ The percentage of DPPH inhibition was calculated using the following formula ^21^:

$$\text{DPPH inhibition}\;\left(\%\right)=\frac{A_{\text{contol}}-A_{\text{sample}}}{A_{\text{control}}}\times 100\%$$

### Alpha-glucosidase inhibitory assay

Briefly, each EEEE sample was dissolved in dimethyl sulfoxide (DMSO) to obtain a concentration of 500 μg/mL. The enzyme solution was prepared by dissolving 1.0 mg of α-glucosidase in a phosphate buffer saline (PBS) (pH 7) containing 200 mg of serum bovine albumin. Before use, 1 mL of the enzyme was pre-diluted 25 times with PBS (pH 7.0). The reaction was initiated by mixing 10 μL of the sample solution (concentration of 500 μg/mL) with 25 μL of 0.04 U/mL α-glucosidase, 50 μL of 0.1 mM PBS (pH 7.0), and 25 μL of p-nitrophenyl-D-glucopyranose (p-NPG). The mixture was incubated at 37°C for 30 minutes. A 100 μL of 0.2 M sodium carbonate (Na _2_CO _3_) was then added to terminate the reaction, and the absorbance of p-nitrophenol was measured at a wavelength of 410 nm using a UV-Vis spectrophotometer (UV-1800, Shimadzu, Japan). ^22^

Positive control was prepared by dissolving acarbose tablets in PBS (pH 7.0) and HCl 2N. To make a 1% acarbose standard solution, the acarbose tablets was diluted in distilled water and 2N HCl at 1:100 ratio. The solution was then centrifuged at 10.000 rpm for 10 minutes and the supernatant was used as a standard. Each test was performed in triplicate, and the inhibitory activity of the extract against α-glucosidase was quantified in % inhibition using the following formula:

$$\%\;\text{inhibition}=\frac{\;A_{\text{control}-}A_{\text{sample}}}{A_{\text{control}}}\times 100\%$$

$A_{\text{control}}$ is the solution absorbance in the absence of the extracts and A _sample_ is the solution absorbance in the presence of the extracts. The IC _50_ value was obtained by plotting the log concentration against the percent (%) inhibition using a regression equation. The IC _50_ represent a concentration inhibiting 50% of the enzyme activity. Based on the IC _50_ values, antioxidant activities are categorized as very strong (IC _50_ value of <50 μg/mL), strong (IC _50_ value of 50-100 μg/mL), weak (IC _50_ of >100 μg/mL), and not active (IC _50_: >250 μg/mL). ^23^

### Data analysis

The IC _50_ of antioxidant activity was determined using a linear regression between concentration (X-axis) and percent inhibition (Y-axis) trough the probit analysis of concentration log data with the probit free radical scavenger (DPPH) percentage. A parametric One-Way Analysis of Variance (ANOVA) was employed to identify significance of differences of the percent inhibition between extracts, followed by Duncan’s multiple range test. All statistical analysis was performed using IBM SPSS software version 25 (IBM Corp., New York, USA), and a *p*-value of ≤0.05 was considered statistically significant.

## Results

### Total phenolic, total flavonoid, and total tannin contents

The result of EEEE phytochemical analysis is presented in Table 1. TPC was expressed in mg GAE/g extract using a standard gallic acid curve (R ^2^ = 0.9237), TFC in mg QE/g extract using a standard quercetin curve (R ^2^ = 0.9282), and TTC in mg TAE/gr extract using a standard tannic acid curve (R ^2^ = 0.9705). The highest TPC was found in the extract of the stem (158.38 mg GAE/g), whereas the lowest was observed in the extract of the leaves (31.35 mg GAE/g). The highest of both TFC (37.91 mg QE/g) and TTC (48.71 TAE/gr) were recorded in the rhizome extract, whereas the lowest were obtained in the leaf extract (TFC: 6.10 QE/g; TTC: 2.13 TAE/gr).

**Table 1.** Total phenolic, total flavonoid, and total tannin content of EEEE.

*E. elatior* Extracts	TPC (mg GAE/g)	TFC (mg QE/g)	TTC (mg TAE/gr)
Rhizome	56.76	37.91	48.71
Stem	158.38	8.31	46.08
Leaf	31.35	6.10	2.13
Flower	127.30	22.79	34.24
Fruit	126.49	22.44	35.82

### Antioxidant activity of EEEE

The result of antioxidant activity of EEEE is presented in Table 2. The most effective radical scavenging activity was shown by the extract of fruit (IC _50_ value of 2.81 μg/mL), whereas the least effective effect was exhibited by the extract of rhizome (IC _50_ value of 188.056 μg/mL). The extracts of stem and leaf showed a very strong antioxidant activity (IC _50_ value of less 50 μg/mL), whereas the extract of flower indicated a strong activity (IC _50_ of 50-100 μg/mL).

**Table 2.** IC _50_ values of EEEE antioxidant activity.

*E. elatior* Extracts	IC _50_ (μg/mL)
Rhizome	188.056 ^*^
Stem	6.966 ^***^
Leaf	19.365 ^***^
Flower	83.095 ^**^
Fruit	2.381 ^***^
Ascorbic acid standard	16.426 ^***^

*** Very strong.

** Strong.

* Weak.

### Alpha-glucosidase inhibition assay of EEEE

**Figure 1.**
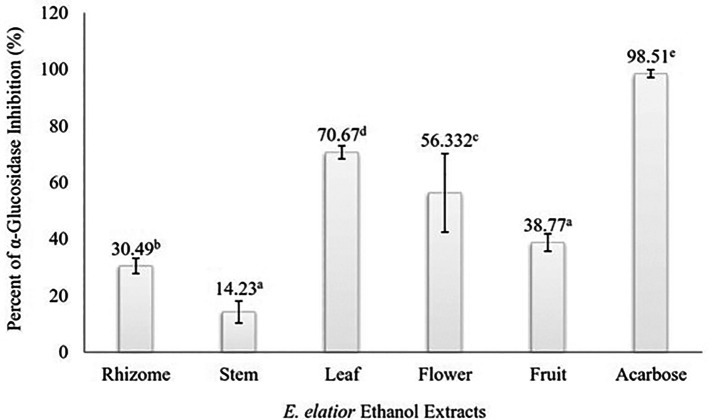
Inhibition of α-glucosidase by EEEE at the concentration of 500 μg/mL. Different superscripts above each bar indicated statistically significant different based on Duncan test (α = 0.05).

Alpha-glucosidase inhibitory activity of EEEE is presented in Figure 1. All the extracts (rhizome, stem, leaf, flower, and fruit) exhibited a statistically significant inhibitory effect on α-glucosidase activity at the concentration of 500 μg/mL ( *p* < 0.05). The result of Duncan test suggested that the percent of inhibition were significantly different among *E. elatior* plant parts, except between the stem and fruit extracts. The extracts of the leaf and flower accounted for more than 50.0% of inhibition, suggesting a promising anti-diabetic potential of *E. elatior.* On the other hand, the lowest inhibition percentage was shown by the stem extract (14.23±3.9%).

The IC _50_ values of EEEE against α-glucosidase are presented in Table 3. The flower extract exhibited the highest IC _50_ values (100 μg/mL), followed by the rhizome, leaf, and fruit extracts. Meanwhile, the lowest IC _50_ values was observed in the extract of stem (5.15 μg/mL), which was comparable to that of acarbose (4.45 μg/mL), highlighting a strong anti α-glucosidase potential of *E. elatior* stem.

**Table 3.** IC _50_ value of EEEE ethanol extracts against α-glucosidase.

*E. elatior* ethanol extracts	IC _50_ (μg/mL)
Rhizome	85.33
Stem	5.15
Leaf	15.25
Flower	100
Fruit	10.07
Acarbose	4.45

## Discussion

The prevalence of type 2 DM (T2DM) is increasing, accounting for approximately 95% of all diabetes cases worldwide. ^1^ This condition can lead to severe complications, such as retinopathy, neuropathy and coronary heart disease if left untreated for a long period of time. ^24^^,^^25^ Therefore, a proper, safe, and effective medication is critical to prevent unexpected complication among sufferers. Medicinal plants have been widely used for the treatment of DM and become a preferable source in the search of novel natural anti-diabetic drugs. In the present study, we evaluated α-glucosidase inhibitory effect of EEEE as a new source of antidiabetic agent.

Phytochemical screening of EEEE indicated the presence of phenolics, flavonoids and tannins. Phenolics represented the most abundant secondary metabolites in EEEE and the content varied among *E. elatior* plant parts. The highest amount was found in the stem extract, followed by flower, fruit, rhizome, and leaf. Phenolics are the most commonly found secondary metabolites in plants synthesized as a response to biotic and abiotic stresses such as temperature, pH, heavy metal stress, and UV radiation. ^26^ Phenolics have been reportedly able to inhibit α-glucosidase activity, justifying its potency for antidiabetic agent. ^27^^,^^28^ Other useful pharmacological properties of phenolic compounds included their ability to treat chronic diseases related to oxidative stress, such as diabetes, cancer, and cardiovascular disorders. ^28^^–^^32^

DPPH radical scavenging assay suggested that the fruit, stem, and leaf extracts of *E. elatior* exhibited a very strong antioxidant activity ( Table 2), which was assumingly attributed to their secondary metabolites such as phenolic and flavonoid content. Phenolics have been reported to facilitate superoxide dismutase (SOD) and glutathione biosynthesis, thus resulting in the prevention and reduction of oxidative stress which has been associated with pancreatic-cell dysfunction exacerbation. ^33^^,^^34^ The ability of phenolic compounds to react with the peroxyl radical (ROO ^-^), donate electron or hydrogen to neutralize free radicals, and act as prooxidant and metal ion chelation has made these compounds as potential antioxidant agents. ^35^^,^^36^ Flavonoids in *E. elatior* also responsible for antioxidant activities by either causing a direct reactive oxygen species (ROS) scavenging, antioxidant enzyme activation, metal chelation, oxidase inhibition, tocopheryl radical reduction, or nitric-oxide-associated oxidative stress attenuation. ^36^ Among all *E. elatior* plant parts evaluated in the present study, the fruits exhibited the most effective antioxidant activity, assumingly associated with the presence of quercetin in the fruits of this plant. ^37^ Antioxidant properties of plant extracts have often been associated with various pharmacological activities including immunomodulatory, antitumor, anti-inflammatory, and antidiabetic potentials. ^12^^,^^38^

We found that all *E. elatior* plant parts significantly inhibited α-glucosidase activity at the concentration of 500 μg/mL. The highest inhibitory percentage was observed in the leaf extract (70.67±2.3%), whereas the lowest was noted in the stem extract (14.23±3.9%). The ability of *E. elatior* leaves to interfere with α-glucosidase activity was assumingly attributed to its chlorogenic compounds that inhibit glucose transporter through their interaction with glucose absorption in the intestine, ^28^ which is similar to the mechanism of action of acarbose, a pharmaceutical drugs used in the treatment of type 2 diabetes. ^39^^–^^41^ In terms of effectivity, the stem extract was found to be the most effective α-glucosidase inhibitor among all *E. elatior* plant parts as it possessed the lowest IC _50_ value (5.15 μg/mL), which was comparable to that of acarbose (4.45 μg/mL). Similar finding was also reported in a previous study, suggesting antihyperglycemic effect of *E. elatior* stem extract *in vivo*. ^32^ This superior α-glucosidase inhibitory effect of the stem was in consistent with the dominant levels of phenolic contents in this part of the plant. Plant-derived polyphenols have been reportedly able to inhibit α-glucosidase activity by interacting with the active sites of this enzyme receptors with a high binding affinity. ^42^

The extract of *E. elatior* flower also exhibited more than 50.0% of α-glucosidase inhibition *in vitro*, suggesting its antidiabetic potential. This finding was in accordance with that of a former study, reporting a high percentage (52.39%) of α-glucosidase inhibition by *E. elatior* flower extract at a concentration of 100 μg/mL *in vitro* and reduced blood glucose level by 57.07% at a concentration of 1000 mg/kg against induced type-2 DM animals model. ^13^ This part of the plant is rich in fiber that might be potential to reduce cholesterol levels and treat various diseases such as hypertension, heart disease, constipation, and diabetes. ^36^ Anti-hyperglycemic activity *E. elatior* flower has been closely related to its antioxidant properties, such as phenolic and flavonoid compounds, ^28^ which have been evidenced to effectively reduce free radicals. ^43^ In addition, alkaloids, flavonoid glucosides, and saponins, which are also found in *E. elatior* flowers, are among other potential hypoglycemic agents that play a crucial role in the prevention of diabetes by hindering α-glucosidase enzyme activity. ^28^^,^^44^^–^^47^ In Indonesian society, young flowers of this plant have been widely used as food ingredients in traditional dishes and empirically used as traditional medicine. ^15^

In addition to the leaves and flowers, the extract of fruit was another *E. elatior* plant part effectively hindering α-glucosidase activity, with percent inhibition of almost 40.0% and IC _50_ value of less than 50 μg/mL. It has been traditionally used as an antidiabetic drug and is rich in anthocyanins constituent. ^36^ Anthocyanins, compounds found in dark colored fruits, has been reported to exhibit anti-inflammatory, antimicrobial, and antidiabetic potentials. ^48^ The synergistic effect of anthocyanins and phenolic compounds is responsible for enzyme inhibitory, antioxidant, and anti-inflammatory activities. ^49^ These findings can lead to future research on the therapeutic use of the *E. elatior* plant in preventing diabetes.

This study possesses several limitations that should be addressed. We employed spectrophotometric method to obtain data, which could have a relatively limited sensitivity and selectivity. Detecting extremely low concentrations of an analyte or distinguishing the analyte from other substances with similar absorption wavelength might be challenging, potentially leading to data bias.

## Conclusion

Ethanol extracts of different *E. elatior* plant parts significantly inhibited α-glucosidase activity *in vitro.* Stem extract showed the most effective α-glucosidase inhibitory effect, highlighting its potential as a future antidiabetic agent. Further study assessing anti α-glucosidase activity of *E. elatior* extracts using non-polar solvents, as well as identification of specific bioactive constituent responsible for α-glucosidase inhibition should be carried out as an effort to develop effective novel anti-diabetic drugs of natural origin.

## Author contributions

Conceptualization: ZZ, SS; data curation: NA, SS, MH; formal analysis: ZZ, NA, SS; funding acquisition: ZZ; investigation: SS, NA; methodology: ZZ, NA; project administration: ZZ; resources: ZZ; software: SS; supervision: ZZ; validation: ZZ, NA, SS; writing – original draft preparation: ZZ, SS, MH; writing – review & editing: ZZ, MH.

## Data Availability

Figshare: ‘In Vitro Alpha-Glucosidase Inhibitory Effect of *Etlingera elatior* Ethanol Extract Growing in Gayo Highland, Aceh Province. https://doi.org/10.6084/m9.figshare.25333411.v1. ^50^ This project contains the following underlaying data:
1. OD value and percent inhibition (%) of EEEE against DPPH radical scavenging.xlsx 2. OD value and percent inhibition (%) of EEEE against α-glucosidase.xlsx Data are available under the terms of the Creative Commons Attribution 4.0 International licence (CC-BY 4.0).
