# Supplementary material for: In Vitro Alpha-Glucosidase Inhibitory Effect of Etlingera Elatior Ethanol Extract Growing in Gayo Highland, Aceh Province, Indonesia
Source: F1000Res. 2024 Sep 4;13:489. Originally published 2024 May 17. [Version 2] doi: 10.12688/f1000research.149029.2 (PMC11487234; doi:10.12688/f1000research.149029.2)
Supplement: Peer review history

# Reviewer report: Reviewer response for version 2

**DOI:** 10.5256/f1000research.171238.r330766

**Published:** 2024-10-17

**Authors:** Dora Dayu Rahma Turista

**Affiliations:** 1 Universitas Mulawarman, Samarinda, East Kalimantan, Indonesia

The introduction has been described sharply. the research method is very clear and detailed so that it makes it easier for readers to understand. The results obtained are in accordance with the method used. The discussion is described in detail by discussing the research with related references. The conclusion is quite concise and clear that reflects the research results.

Is the work clearly and accurately presented and does it cite the current literature?

Yes

If applicable, is the statistical analysis and its interpretation appropriate?

Yes

Are all the source data underlying the results available to ensure full reproducibility?

Yes

Is the study design appropriate and is the work technically sound?

Yes

Are the conclusions drawn adequately supported by the results?

Yes

Are sufficient details of methods and analysis provided to allow replication by others?

Yes

Reviewer Expertise:

Natural Resources

I confirm that I have read this submission and believe that I have an appropriate level of expertise to confirm that it is of an acceptable scientific standard.

# Reviewer report: Reviewer response for version 2

**DOI:** 10.5256/f1000research.171238.r320734

**Published:** 2024-09-13

**Authors:** Arif Nur Muhammad Ansori

**Affiliations:** 1 Universitas Airlangga, Surabaya, East Java, Indonesia; 2 Division of Research and Development, Jalan Tengah, Surabaya, Indonesia

The revised manuscript provides a well-structured and comprehensive evaluation of the alpha-glucosidase inhibitory activity and antioxidant properties of *Etlingera elatior* ethanol extract. The study presents significant data on the phytochemical content and its implications in diabetes treatment. The objectives are clear, the methodology is sound, and the results are well presented.

Is the work clearly and accurately presented and does it cite the current literature?

No

If applicable, is the statistical analysis and its interpretation appropriate?

I cannot comment. A qualified statistician is required.

Are all the source data underlying the results available to ensure full reproducibility?

Yes

Is the study design appropriate and is the work technically sound?

Yes

Are the conclusions drawn adequately supported by the results?

Yes

Are sufficient details of methods and analysis provided to allow replication by others?

Yes

Reviewer Expertise:

NA

I confirm that I have read this submission and believe that I have an appropriate level of expertise to confirm that it is of an acceptable scientific standard.

# Reviewer report: Reviewer response for version 1

**DOI:** 10.5256/f1000research.163430.r288676

**Published:** 2024-08-29

**Authors:** Arif Nur Muhammad Ansori

**Affiliations:** 1 Universitas Airlangga, Surabaya, East Java, Indonesia; 2 Division of Research and Development, Jalan Tengah, Surabaya, Indonesia

First of all, this manuscript is good and well-written. However, I may require some comments on the following issues. Basically, this manuscript is acceptable. 

 Language can be improved for the readability of the paper. 

 Title: The title is easy to follow and no mistakes.

 Abstract: This section was well-written and easy to understand. In addition, the study's objective and the state of the art of the study is clear. Furthermore, the keywords should represent the study.

 Discussion: To develop a systematic discussion, the authors should mention the limitation(s) of the study.

 Conclusion: In this part of the paper, the authors have established the conclusion of this study.

 References: Many references are out of guidelines. Please kindly check the references again, do not include the old references, and check the abbreviation of the journals/proceedings. Please mention many high-quality related publications. References number 14, 15, 16, 24, and 33 should be changed.

Is the work clearly and accurately presented and does it cite the current literature?

No

If applicable, is the statistical analysis and its interpretation appropriate?

I cannot comment. A qualified statistician is required.

Are all the source data underlying the results available to ensure full reproducibility?

Yes

Is the study design appropriate and is the work technically sound?

Yes

Are the conclusions drawn adequately supported by the results?

Yes

Are sufficient details of methods and analysis provided to allow replication by others?

Yes

Reviewer Expertise:

NA

I confirm that I have read this submission and believe that I have an appropriate level of expertise to confirm that it is of an acceptable scientific standard, however I have significant reservations, as outlined above.

# Response

**Published:** 2024-08-30

**Authors:** zumaidar zumaidar

**Affiliations:** Biology, Universyitas Syiah Kuala, Banda Aceh, Indonesia, Indonesia

Thank you for reviewing our manuscript. The comments and suggestions have improved the quality of our current manuscript significantly. 

 References: Many references are out of guidelines. Please kindly check the references again, do not include the old references, and check the abbreviation of the journals/proceedings. Please mention many high-quality related publications. References number 14, 15, 16, 24, and 33 should be changed.

 RESPONSE: Thank you for the suggestions.

 1. We agree to exclude reference number 9 (old reference) as it is not strongly relevant to our study. Since it supports general information in the introduction, it will not affect any specific content in our manuscript.

 2. We sincerely appreciate the suggestion to exclude older references from the manuscript. However, we believe that several of these references are crucial to our study. Some provide essential information on the phytochemical content and pharmacological activities of *Etlingera elatior*, which serve as the foundational basis for our research. Therefore, we have chosen to retain these references, especially for reference. 

 3. We appreciate the reviewer's suggestion regarding the exclusion of references number 14, 15, 16, 24, and 33. However, we believe these references are highly relevant to our study as they provide essential information that directly supports our findings. Therefore, we kindly request to retain these references in the manuscript.
